# Predilation Ballooning in High Thrombus Laden STEMIs: An Independent Predictor of Slow Flow/No-Reflow in Patients Undergoing Emergent Percutaneous Coronary Revascularization

**DOI:** 10.1155/2023/4012361

**Published:** 2023-01-06

**Authors:** Rajesh Kumar, Danish Qayyum, Ifikhar Ahmed, Lajpat Rai, Ayaz Mir, Romana Awan, Ali Bin Naseer, Abdul Basit, Jawaid Akbar Sial, Tahir Saghir, Nadeem Qamar, Musa Karim

**Affiliations:** ^1^National Institute of Cardiovascular Diseases (NICVD), Karachi, Pakistan; ^2^National Institute of Cardiovascular Diseases (NICVD), Hyderabad, Pakistan

## Abstract

**Background:**

Distal embolization due to microthrombus fragments formed during predilation ballooning is considered one of the possible mechanisms of slow flow/no-reflow (SF/NR). Therefore, this study aimed to compare the incidence of intraprocedure SF/NR during the primary percutaneous coronary intervention (PCI) in patients with high thrombus burden (≥4 grade) with and without predilation ballooning for culprit lesion preparation. *Methodology*. This prospective descriptive cross-sectional study included patients with a high thrombus burden (≥4 grades) who underwent primary PCI. Propensity-matched cohorts of patients with and without predilation ballooning in a 1 : 1 ratio were compared for the incidence of intraprocedure SF/NR.

**Results:**

A total of 765 patients with high thrombus burden undergoing primary PCI were included in this study. The mean age was 55.75 ± 11.54 years, and 78.6% (601) were males. Predilation ballooning was conducted in 346 (45.2%) patients. The incidence of intraprocedure SF/NR was significantly higher (41.3% vs. 27.4%; *p* < 0.001) in patients with predilation ballooning than in those without preballooning, respectively. The incidence of intraprocedure SF/NR also remained significantly higher for the predilation ballooning cohort with an incidence rate of 41.3% as against 30.1% (*p*=0.002) for the propensity-matched cohort of patients without predilation ballooning with a relative risk of 1.64 (95% CI: 1.20 to 2.24). Moreover, the in-hospital mortality rate remained higher but insignificant, among patients with and without predilation ballooning (8.1% vs. 4.9%; *p*=0.090).

**Conclusion:**

In conclusion, predilation ballooning can be associated with an increased risk of incidence of intraprocedure SF/NR during primary PCI in patients with high thrombus burden.

## 1. Introduction

ST-segment elevation myocardial infarction (STEMI) is the most common clinical manifestation of acute myocardial infarction (AMI). Primary percutaneous coronary intervention (pPCI) within a 12 hour window period is the recommended management strategy for patients with acute STEMI. Early restoration of antegrade blood flow is the primary target of pPCI in infarct-related arteries [1]. However, the occurrence of the slow-flow or no-reflow phenomenon jeopardized the benefits of pPCI. Even with such an effective management strategy, the optimal reperfusion and achievement of target Thrombolysis in Myocardial Infarction (TIMI) flow grade III is not possible in all STEMI patients. The coexistence of a patent epicardial coronary artery and the state of myocardial tissue hypoperfusion is termed “slow-flow/no-reflow (SF/NR)” [2]. The etiology and exact mechanism behind this dual state phenomenon are not very clear, and various proposed etiologic aspects include distal coronary embolization, inflammatory response, vasospasm, capillary obstruction, endothelial swelling, myocardial edema, and ischemia-reperfusion injury [3, 4].

According to recent studies, in the contemporary era, the occurrence of SF/NR ranges from 4% to 30% after pPCI [2, 5–11]. Various predisposing factors have been identified, and several explanative mechanisms have been proposed for SF/NR phenomenon along with approaches to overcome SF/NR in clinical practice. Among various clinical factors, thrombosis burden (≥4 grade) has been also reported to be an important factor strongly associated with the development of SF/NR during pPCI [6, 10–13]. Thrombosuction or predilection has been reported to be associated with an increased risk of SF/NR in a few studies [13]. We postulate distal embolization due to microthrombus fragments formed during predilation ballooning as a possible mechanism of SF/NR among patients with a high thrombus burden. Hence, this study aimed to assess the effect of predilation ballooning on the incidence of intraprocedure SF/NR during primary PCI in patients with a high thrombus burden (≥4 grade).

## 2. Materials and Methods

This prospective descriptive cross-sectional study was approved by the ethical review committee (ERC) of the National Institute of Cardiovascular Diseases (NICVD), Karachi, Pakistan (ERC-30/2020). The study sample consisted of consecutive patients diagnosed with STEMI undergoing pPCI during the study duration of August 2020 to July 2021. Inclusion criteria were patients diagnosed with STEMI, fulfilling the criteria of pPCI, and angiographic evidence of high thrombus burden (≥4 grade). Patients who underwent manual thrombus aspiration were excluded. Before inclusion, verbal informed consent was obtained from all patients. All primary PCI procedures were performed by clinical practice guidelines for the management of STEMI.

A structured proforma was used to collect the demographic data, clinical characteristics, and PCI procedural details of the study cohort. The periprocedure occurrence of an episode of SF/NR was defined as SF/NR based on low-anterograde coronary flow (TIMI flow grade of <III) in the infarct-related artery. The thrombus grade was categorized as grade G0 to G5. High thrombus burden was taken as ≥4 grade which is thrombus in more than half of the vessel diameter with total vessel occlusion. The decision of stenting was made by the primary operator based on distal edge visualization of the culprit segment after guidewire Dottering or preballooning. Angiographic films were assessed for SF/NR by independent consultant cardiologists blinded to the predilation ballooning status. As per the guideline recommendations and institutional primary PCI protocol, all patients were premedicated with unfractionated heparin and dual antiplatelet therapy (DAPT) along with a bolus dose of glycoprotein inhibitors (IIb/IIIa). The use of ticagrelor was limited to patients with diabetes, high-risk anatomy, or stent thrombosis, while clopidogrel and aspirin were the most commonly used DAPT due to cost-effectiveness.

Considering the statistical implications of significant differences in clinical characteristics of cohorts with and without predilation ballooning for the comparison of the incidence rate of SF/NR, propensity matching was performed in a 1 : 1 ratio of predilation ballooning and non-predilation ballooning cohorts. Propensity matching was performed using R software version 3.6.1 with the help of the “MatchIt” package. Clinical characteristics used for propensity matching were age (years), gender, total ischemic time (minutes), systolic blood pressure (mmHg), heart rate (bpm), random blood sugar (mg/dL), Killip class, intubation status, cardiac arrest, diabetes mellitus, hypertension, smoking, history of prior PCI, history of stroke, left ventricular end-diastolic pressure (mmHg), left ventricular ejection fraction (%), use of intra-aortic balloon pump, number of diseased vessels, infarct-related artery, preprocedure and postprocedure TIMI flow grade, vessel diameter, and length of the lesion. Two groups were compared by applying an independent sample *t*-test/Mann–Whitney *U* test or Fisher's exact test/chi-square test with the help of IBM SPSS version 21. The criteria for statistical significance of the difference between men and women were *p* ≤ 0.05 throughout the analysis.

## 3. Results

A total of 765 patients with high thrombus burden undergoing pPCI were included in this study. The mean age was 55.75 ± 11.54 years, and 78.6% (601) were males. Predilation ballooning was conducted in 346 (45.2%) patients, while Dottering was conducted in 241 (31.5%) patients and direct stenting in the remaining 178 (23.3%) patients after visualization of the distal edge of the culprit segment after guidewire. The predilation ballooning cohort was observed to be significantly different in terms of age distribution (56.95 ± 11.3 years vs. 54.77 ± 11.65 years; *p*=0.009), median total ischemic time (375 [250–570] vs. 342 [240–460]; *p*=0.021), Killip class III/IV (18.2% vs. 11.9%), presence of diabetes (43.1% vs. 36%; *p* = 0.048), mean left ventricular end-diastolic pressure (20 ± 7.6 mmHg vs. 19 ± 6.7 mmHg; *p*=0.046), mean stent diameter (3.4 ± 0.4 mm vs. 3.5 ± 0.3 mm; *p* < 0.001), mean total length of stent implanted (29.8 ± 12.2 mm vs. 26.3 ± 11.3 mm; *p* < 0.001), and three-vessel diseases (35% vs. 26.5%) compared to the patients without preballooning. The incidence of intraprocedure SF/NR was significantly higher (41.3% vs. 27.4%; *p* < 0.001) in the predilation ballooning cohort than in non-predilation ballooning cohort, respectively (Table 1).

To minimize the confounding effect of various clinically and statistically significant variables on the incidence of intraprocedure SF/NR, the predilation ballooning cohort was compared to a propensity-matched non-predilation ballooning cohort (Table 2).

The incidence of intraprocedure SF/NR remained significantly higher for the predilation ballooning cohort with an incidence rate of 41.3% against 30.1% (*p*=0.002) for the non-predilation ballooning cohort with the relative risk of 1.64 [95% CI: 1.20 to 2.24]. Moreover, the in-hospital mortality rate remained higher but insignificant, among the predilation ballooning cohort (8.1% vs. 4.9%; *p*=0.090) compared to the non-predilation ballooning cohort.

## 4. Discussion

Predilation in patients with a high thrombus burden increases the risk of intraprocedure SF/NR with a possible mechanism of distal embolization due to microthrombus fragments which can be worsened during predilation ballooning while preparing the thrombus laden culprit lesion during pPCI. Hence, we conducted this study to assess the effect of predilation ballooning on the incidence of intraprocedure SF/NR during primary PCI in patients with a high thrombus burden (≥4 grade). In our study, the patient cohort with predilation ballooning had a significantly higher incidence of intraprocedure SF/NR than in the cohort without preballooning. However, the construction of both cohorts was significantly different in terms of various clinical characteristics such as age distribution, total ischemic time, Killip class III/IV, presence of diabetes, LVEDP, stent diameter, and total length of stent implanted, and three-vessel disease. Hence, we constructed a propensity-matched cohort of non-predilation ballooning patients and compared it with the predilation ballooning cohort for the incidence of intraprocedure SF/NR, and we observed that even after matching for differences in clinical characteristics, the incidence of intraprocedure SF/NR remained significantly higher among the predilation ballooning cohort with the incidence rate of 41.3% as against 30.1% (*p*=0.002) for non-predilation ballooning cohort with a relative risk of 1.64 [95% CI: 1.20 to 2.24].

Following our observations, several studies have shown that a high thrombus burden is a prominent risk factor for SF/NR phenomena [14, 15]. In a study of 794 patients with AMI, high thrombus burden emerged as an independent predictor of SF/NR after emergency PCI [16]. Another study indicated that the percentage of individuals having high thrombus burden in the SF/NR cohort was higher compared to the cohort with normal flow [6]. In one way or another, the risk of no reflow was significantly correlated with preprocedure TIMI flow, grade I, collateral flow, multivessel involvement, and high thrombus burden. Furthermore, TIMI flow and thrombus burden were the two most commonly related covariates to SF/NR compared to other covariates. Patients with large infarct size along with high thrombus burden [17], decreased TIMI flow, and absence of collateral flow [18] are more likely to develop SF/NR [19].

Microembolization following percutaneous coronary intervention has been associated with a high thrombus burden in magnetic resonance imaging (MRI) study within one year of acute myocardial infarction reported by Amabile et al. [20] Data from the recent studies also identified thrombus burdens of ≥4 before PCI as among some of the independent predictors of SF/NR [10, 11, 21, 22]. In a typical clinical setting, extended ischemic time is generally accompanied by a high thrombus burden, as late reperfusion results in more erythrocytes and more thrombus accumulation. Prolonged ischemia can also cause capillary integrity to deteriorate, as well as polymorphonuclear cell plugging and edema in the myocardial cell and capillary bed [13, 21]. Particularly, in the process of balloon dilatation, red thrombi form fragments and cause distal embolization by washing into the distal end of the artery, consequently leading to reduced perfusion of myocardial tissue [13, 18, 21, 23–26]. Consequently, microcirculatory dysfunction worsens myocardial reperfusion injury and increases the risk of SF/NR and adverse cardiovascular events. Okamura et al. [27] reinforced these observations for patients who underwent PCI with the finding of multiple embolic particles in a study using Doppler guidewires. Embolization has been observed to be associated with an irreversible reduction of myocardial blood flow with the blockage of up to 50% of the coronary capillaries [28]. In addition to fragmentation of the thrombus, there can be other multiple possible causes for predilatation induced SF/NR, such as microembolization of older and more mature thrombi, which may become worse with acute plaque rapture and may result into more severe infarct-related artery occlusion and higher coronary wedge pressure [29]. Hence, in this study, we have taken the total ischemic time as one of the main parameters of propensity matching between the two groups.

This is the first study on the impact of balloon dilation on the incidence of SF/NR in patients with high thrombus burden. The observational nature of the study is one of the major limitations of this study. Secondly, data on myocardial blush grade (MBG) were not available for comparison between the two cohorts. A randomized study of predilation ballooning in high thrombus burden can provide conclusive evidence in the context of SF/NR.

## 5. Conclusion

In conclusion, pre-dilation ballooning can be associated with an increased risk of incidence of intraprocedure SF/NR during primary PCI in patients with a high thrombus burden. Considering the prognostic significance of SF/NR, it is important to avoid predilation ballooning and limit the hardware used during primary PCI of patients with high thrombus burden to avoid SF/NR.

## Figures and Tables

**Table 1:** Demographic, clinical, and angiographic characteristics and postprocedure in-hospital complications and outcomes stratified by predilation ballooning status.

	Preballooning		*p* value
	Not done	Done	
Total (*N*)	419 (54.8%)	346 (45.2%)	—
Gender			
Female	20.3% (85)	22.8% (79)	0.393
Male	79.7% (334)	77.2% (267)	
Age (years)	54.77 ± 11.65	56.95 ± 11.3	0.009
Total ischemic time (minutes)	342 [240–460]	375 [250–570]	0.021
Systolic blood pressure (mmHg)	129.9 ± 25.3	131.3 ± 27.1	0.449
Heart rate (bpm)	83.2 ± 21.5	86.8 ± 21.8	0.021
Random glucose level (mg/dL)	155 [128–211]	160 [132–212]	0.301
Killip class			
I	76.4% (320)	67.1% (232)	0.021
II	11.7% (49)	14.7% (51)	
III	6.4% (27)	11.6% (40)	
IV	5.5% (23)	6.6% (23)	
Cardiac arrest	6% (25)	7.8% (27)	0.315
Comorbid conditions			
Diabetes mellitus	36% (151)	43.1% (149)	0.048
Hypertension	56.1% (235)	63% (218)	0.053
Smoking	32.2% (135)	27.5% (95)	0.153
Prior PCI	7.9% (33)	9.5% (33)	0.415
History of CVA/TIA	2.1% (9)	1.4% (5)	0.470
IABP used	5.5% (23)	7.5% (26)	0.255
LV ejection fraction (%)	39.6 ± 8.9	38.9 ± 9.5	0.276
LV end-diastolic pressure (mmHg)	19 ± 6.7	20 ± 7.6	0.046
Number of vessels involved			
Single vessel disease	40.8% (171)	30.9% (107)	0.008
Two vessel disease	32.7% (137)	34.1% (118)	
Three vessel disease	26.5% (111)	35% (121)	
Culprit coronary artery			
Left main	1% (4)	2.6% (9)	0.119
LAD; proximal	34.6% (145)	32.7% (113)	
LAD; nonproximal	17.4% (73)	21.1% (73)	
Left circumflex artery	9.8% (41)	12.1% (42)	
Right coronary artery	37.2% (156)	31.5% (109)	
Pre-procedure TIMI flow			
0	82.6% (346)	85.5% (296)	0.265
I	17.4% (73)	14.5% (50)	
II	0% (0)	0% (0)	
III	0% (0)	0% (0)	
Mean stent diameter	3.5 ± 0.3	3.4 ± 0.4	<0.001
Total stent length	26.3 ± 11.3	29.8 ± 12.2	<0.001
Intraprocedure slow flow/no-reflow	27.4% (115)	41.3% (143)	<0.001
Final TIMI III flow	85.4% (358)	84.4% (292)	0.686
In-hospital complications			
Stroke	0.7% (3)	0% (0)	0.115
Arrhythmia	5% (21)	6.9% (24)	0.260
Cardiogenic shock	4.3% (18)	4% (14)	0.864
Stent thrombosis	3.3% (14)	2% (7)	0.267
Contrast induced nephropathy	8.8% (37)	12.4% (43)	0.106
In-hospital mortality	4.1% (17)	8.1% (28)	0.018

PCI = percutaneous coronary intervention, CVA = cerebrovascular accident, TIA = transient ischemic attack, IABP = intraaortic balloon pump, LV = left ventricular, LAD = left anterior descending artery, and TIMI = thrombolysis in myocardial infarction.

**Table 2:** Demographic, clinical, and angiographic characteristics and postprocedure in-hospital complications and outcomes' comparison between propensity-matched cohorts of patients with and without predilation ballooning in 1 : 1 ratio.

	Preballooning		*p* value
	Not done	Done	
Total (*N*)	346	346	—
Gender			
Female	22% (76)	22.8% (79)	0.784
Male	78% (270)	77.2% (267)	
Age (years)	55.55 ± 11.51	56.95 ± 11.3	0.108
Total ischemic time (minutes)	360 [252–480]	375 [250–570]	0.239
Systolic blood pressure (mmHg)	130.4 ± 25.8	131.3 ± 27.1	0.670
Heart rate (bpm)	84.5 ± 21.6	86.8 ± 21.8	0.159
Random glucose level (mg/dL)	155 [128–215]	160 [132–212]	0.340
Killip class			
I	74% (256)	67.1% (232)	0.169
II	12.1% (42)	14.7% (51)	
III	7.5% (26)	11.6% (40)	
IV	6.4% (22)	6.6% (23)	
Cardiac arrest	6.6% (23)	7.8% (27)	0.557
Comorbid conditions			
Diabetes mellitus	39% (135)	43.1% (149)	0.279
Hypertension	58.4% (202)	63% (218)	0.213
Smoking	30.3% (105)	27.5% (95)	0.402
Prior PCI	8.7% (30)	9.5% (33)	0.692
History of CVA/TIA	1.2% (4)	1.4% (5)	0.737
IABP used	5.8% (20)	7.5% (26)	0.360
LV ejection fraction (%)	39.3 ± 8.9	38.9 ± 9.5	0.608
LV end-diastolic pressure (mmHg)	19.4 ± 6.9	20 ± 7.6	0.275
Number of vessels involved			
Single vessel disease	36.7% (127)	30.9% (107)	0.189
Two vessel disease	33.8% (117)	34.1% (118)	
Three vessel disease	29.5% (102)	35% (121)	
Culprit coronary artery			
Left main	1.2% (4)	2.6% (9)	0.352
LAD; proximal	33.8% (117)	32.7% (113)	
LAD; nonproximal	19.9% (69)	21.1% (73)	
Left circumflex artery	9.2% (32)	12.1% (42)	
Right coronary artery	35.8% (124)	31.5% (109)	
Preprocedure TIMI flow			
0	84.1% (291)	85.5% (296)	0.596
I	15.9% (55)	14.5% (50)	
II	0% (0)	0% (0)	
III	0% (0)	0% (0)	
Mean vessel diameter	3.5 ± 0.3	3.4 ± 0.4	0.044
Total stent length	27.8 ± 11.5	29.8 ± 12.2	0.030
Intraprocedure slow flow/no-reflow	30.1% (104)	41.3% (143)	0.002
Final TIMI III flow	84.4% (292)	84.4% (292)	>0.999
In-hospital complications			
Stroke	0% (0)	0% (0)	-
Arrhythmia	5.5% (19)	6.9% (24)	0.431
Cardiogenic shock	5.2% (18)	4% (14)	0.469
Stent thrombosis	4% (14)	2% (7)	0.121
Contrast induced nephropathy	9.2% (32)	12.4% (43)	0.179
In-hospital mortality	4.9% (17)	8.1% (28)	0.090

PCI = percutaneous coronary intervention, CVA = cerebrovascular accident, TIA = transient ischemic attack, IABP = intraaortic balloon pump, LV = left ventricular, LAD = left anterior descending artery, and TIMI = thrombolysis in myocardial infarction.

## Data Availability

The data materials used to support the findings of this study can be obtained upon reasonable request.
